# Walkability and urban built environments—a systematic review of health impact assessments (HIA)

**DOI:** 10.1186/s12889-023-15394-4

**Published:** 2023-03-17

**Authors:** Joachim Westenhöfer, Elham Nouri, Merle Linn Reschke, Fabian Seebach, Johanna Buchcik

**Affiliations:** Competence Center Health and Department Health Sciences, Hamburg University of Applied Sciences, Ulmenliet 20, 21033 Hamburg, Germany

**Keywords:** Health impact assessment, Built environment, Walkability

## Abstract

**Background:**

Urban environments are important determinants of human health. The term walkability summarizes features of the urban built environment that promote walking and other types of physical activity. While the beneficial effects of active and public transport have been well established, the health impact of other features of walkability are less well documented.

**Methods:**

We conducted a systematic review of health impact assessments (HIAs) of walkability. Studies were identified through PUBMED and Science Direct, from two German websites related to urban health and reference tracking. Finally, 40 studies were included in the present review. We applied qualitative thematic analysis to summarize the major results from these studies.

**Results:**

Most of the HIAs (*n* = 31) reported the improvement of health or health behaviour resulting from an investigated project or policy. However, three HIAs reported a lack of improvement or even a decrease of health status. In parallel, 13 HIAs reported a gain in economic value, whereas one reported a lack or loss of economic effects. Moreover, three HIAs reported on social effects and six HIAs gave additional recommendations for policies or the implementation of projects or HIAs.

**Conclusions:**

Most HIAs investigate the impact of increasing active or public transport. Other features of walkability are less well studied. With few exceptions, HIAs document beneficial impacts of improving walkability on a variety of health outcomes, including reductions of mortality and non-communicable diseases.

**Supplementary Information:**

The online version contains supplementary material available at 10.1186/s12889-023-15394-4.

## Background

Human health is influenced by a variety of determinants including factors related to the environment where people live [1]. Urbanization has become one of the global megatrends that characterize the current development of mankind. At the beginning of the 20th century, only about 10 percent of the world’s population were living in urban areas. In 2015, this percentage increased to about 54%, and is predicted to reach 60% in 2030 and 66% by 2050 [2].

Urbanization more often offers health advantages in comparison with rural areas, as the basic infrastructure relevant for health such as water, sanitation and housing are generally more developed. In addition, health services and facilities appear to be more available in cities than in rural areas. However, cities may also cause relative disadvantages for health, e.g. crowded living and stressing working conditions, higher rates of crime and violence, sedentary life styles, reduced physical activity, and, additionally, the urban food environment may contribute to the rise of non-communicable diseases [2].

However, many decisions that impact human health are made outside the health sector [3, 4]. For example, environmental changes resulting from the intensification of agriculture, industrialization and increasing energy use are considered as important sources of health problems [5]. Decisions about the quality of social services, housing, employment opportunities or public transport are among many others key influences on health [6], and are again usually made outside the health sector. This has led the WHO to extend the ideas of healthy public policy, already formulated in the Ottawa Charta of Health Promotion to the principle of “Health in All Policies” [7]. Yet, considering not only reduction of health risks, but also enhancing health promoting potentials in urban development and urban planning seems not to be implemented systematically on a large scale [6].

Health Impact Assessment (HIA) is an approach to bring health considerations into urban development and urban planning. HIA has been defined as “… a combination of procedures, methods and tools by which a policy, program or project may be judged as to its potential effects on the health of a population, and the distribution of those effects within the population” [4]. One aim is to produce recommendations for decision makers and stakeholders for “… maximizing the proposal’s positive health effects and minimizing its negative health effects” [8]. HIAs have been conducted in all regions of the world, and the majority of HIA practitioners expect an increased use in Australia, East Asia and Pacific, Europe and North America [9].

The impact of urban environment on physical activity has received some consideration during the last decades. Globally, physical inactivity has been accounted for the fourth leading cause of mortality, after high blood pressure, tobacco use and high blood glucose, contributing to 6 percent of worldwide deaths [10]. The Global Burden of Disease study is using a sophisticated hierarchical model of risk factors including physical inactivity as a level 2 risk factor [11]. Recently, results from this study indicated that, globally seen, close to 1 million deaths in 2019 were attributable to physical inactivity [11]. In addition, physical inactivity is considered as a major risk factor for non-communicable disease, particularly cardio-vascular diseases, diabetes mellitus type 2, and several types of cancer [12]. Moreover, physical activity contributes to the maintenance of healthy weight and to the prevention of overweight and obesity [12] which in turn is a major risk factor for the mentioned NCDs [13]. On the other hand, the beneficial effects of physical activity on all-cause mortality [14–16], the incidence of cardiovascular health, diabetes, several types of cancer [17], and mental health [18, 19] have been well documented.

In relation to physical activity two concepts for the urban environment have received considerable attention and generated some research: active transport and walkability. Active transport comprises walking or cycling for the purpose of reaching a destination such as school, workplace, or a shop [20]. Walkability summarizes attributes of the urban built environment that encourage and/or enable more walking [21–23]. The original concept of walkability was developed in the 1990s in US transportation research and has focused on walking for transportation [24]. As this concept was adopted by physical activity and public health researchers and practitioners, it was extended to include walking for transportation and recreational purposes as well as other types of physical activity, e.g. biking [24]. Hence, walkability has extended beyond walking to generally promoting physical activity in communities, urban neighbourhoods and larger urban areas [25]. A review [23] concludes that there is sufficient evidence that the proximity to potential destinations, aesthetic qualities – the attractiveness of the environment -, mixed land use, residential density within neighbourhoods, sidewalks and connectivity are attributes of the built environment that correlate with increased walking. Recently, a more comprehensive framework of walkability has been suggested [26, 27] that incorporates nine dimensions of the built environment, namely connectivity, diversity of land-use, residential density, traffic safety, surveillance (how well traveling in the street can be seen from surrounding houses and businesses), parking (less parking encourages more walking), experience (including e.g. aesthetics), greenspace and community (social interaction and participation).

The health impacts of active transport have been intensively studied and a systematic review provides strong evidence that active transport provides substantial net health benefits even if negative health impacts like accidents and exposure to air pollution are taken into account [14, 20].

**Fig. 1:**
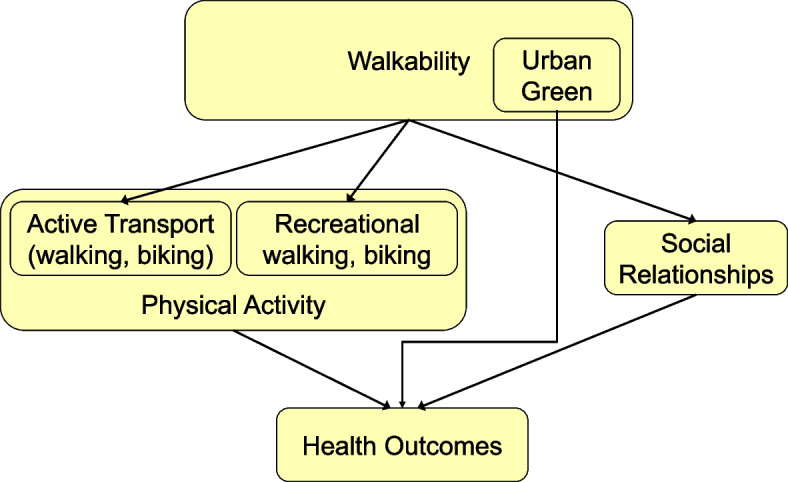
Potential major pathways of the health impact of walkability, own presentation

To the best of our knowledge up to now no systematic review is available to summarize the evidence on the health impacts of walkability conceptualized as detailed above as characteristics of the urban built environment. We hypothesize that the walkability of urban environments may affect health outcomes via several pathways (see Fig. 1): walkability may result in more physical activity either by improving active transport or by encouraging recreational activities including deliberate exercising. In addition, green space improves health by encouraging more physical activity and by other effects, e.g. lower distress and better mental health [28] and the better walkability of the built environment could promote and result in improved social relationships [29] that in turn impact human health.

The objective of this paper is to conduct and report on a systematic review of health impact assessments (HIAs) of projects, policies or programmes that aim to improve the walkability of urban built environments.

Particularly, we aim at answering the following research questions related to such HIAs:

- Which types of projects, policies or programmes related to walkability in urban development have been investigated in HIAs?
- Which methods were used to assess the health impacts? Which data sources were used, and which analytical models were applied to assess health impacts?
- Which health impacts (e.g. changes in mortality, incidence or prevalence of diseases, quality of life) have been identified related to improvements of walkability?
- How and by whom are HIAs of walkability implemented in practice?

## Methods

This systematic review was designed based on the PRISMA 2020 guidelines [30]. We considered the following definition of walkability as the dividing line for identification of eligible articles in the current systematic review: Walkability summarizes attributes of the urban built environment that encourage and/or enable more walking or other types of physical activity in communities, urban neighbourhoods and larger urban areas.

In order to include a wide range of studies, no specific preference for a definition of Health Impact Assessment was considered.

### Data sources and search strategy

We searched PubMed and ScienceDirect databases with the purpose of incorporating international studies as well as the websites of two German associations “Stadt-und-Gesundheit” (City and Health; http://stadt-und-gesundheit.de) and “Akademie für Raumentwicklung in der Leibniz-Gemeinschaft” (ARL – Academy for Territorial Development in the Leibniz Association; https://www.arl-net.de) to identify research reports focusing on spatial planning particularly in the German context. The databases were searched thoroughly in November 2020, and an additional search of PubMed and Science Direct was also conducted in the end of 2021 to update the study pool. We operated the advanced search in PubMed and ScienceDirect using the search terms in Table 1. The two German databases did not offer an advanced search tool. Therefore, an adoption of the strategy for these two sources was necessary. This involved a title screening of all listed papers and reports. We included all papers that mentioned “Health Impact Assessment” (the German equivalent term “Gesundheitsfolgenabschätzung” respectively) or “Walkability” in the title. As the term Walkability was rarely used in the titles of the reports on the two German databases, we accepted the German terms for mobility/mobile, physical activity, walking (distance), transport (ation), walkable, and pedestrian as potential equivalents. For the same reason, we accepted papers with a title suggesting a health outcome related to walkability (e.g. increased walking). The details of identified articles and search terms for each of databases are provided in the Table 1. In addition, reference lists of papers included during the selection process were screened for additional papers that might be relevant.

**Table 1:** Search strategy and identified articles in each database

Database	Search terms	Hits
PubMed	(Health Impact Assessment) AND (Walkability)	24
ScienceDirect	"Health Impact Assessment" AND ‘’Walkability’’	287
Akademie für Raumentwicklung in der Leibniz-Gemeinschaft	Titles suggesting assessment of health impacts of walkability	451
Stadt-und-Gesundheit	Titles suggesting assessment of health impacts of walkability	87

### Inclusion and exclusion criteria

Regarding selection criteria, any peer-reviewed publications evaluating a real or projected (modelled) health impact or health outcomes of a policy, programme or project that intended to change an aspect of walkability in an urban environment were eligible for the review, with the exception of short communications, published abstracts and conference contributions. Furthermore, the eligible articles were published in English or German and after the year 2010. We decided not to include grey literature because we believe that the peer-review process provides an important quality assurance and therefor enhances the credibility of the research findings. In addition, identification of relevant articles via databases of the peer-reviewed scientific literature seems more transparent and replicable than a comprehensive open search using a wide variety of search engines. Details of inclusion and exclusion criteria are available in Table 2.

**Table 2:** Inclusion and exclusion criteria for article selection

Inclusion criteria	Exclusion criteria
Subject:	Subject:
• Real or projected (modelled) health outcomes (e.g. mortality, incidence or prevalence of diseases) of º a policy, programme, or project that º intended to improve or actually improved a feature of the built environment related to walkability º within an urban area	• Studies reporting only on the association between features of the built environment and health outcomes • Studies reporting on health outcomes or health impact of changes of active transport (AT) or physical activity (PA) in general without reference to specific changes of the built environment that could effect such changes of AT or PA • Studies addressing changes in specific settings (e.g. clinical setting as a hospital, school, or kindergarden) but not generally in an urban geographic area
Type of publication:	Type of publication:
• Original publications (journal articles) • Book chapters • Review articles	• Editorials, short communications, abstracts, and conference contributions
Language:	Language:
• English • German	• Any language other than English or German
Year of publication:	Year of publication:
• 2010—2021	• Publication before 2010

### Screening, data extraction and analysis

Title screening was done by one reviewer. In case of any uncertainty, the decision was made after discussion with another author. For the remaining records, abstract screening was done independently by two reviewers. Any disagreement between the reviewers was resolved by discussion, in some rare cases by including additional authors of the present paper.

The final selection step was the review of the full text of remaining articles by two authors. Every step of the review process was discussed during regular meetings in order to clarify uncertainties and challenges related to selection process. In case of disagreement of authors on the eligibility of articles, a third reviewer conducted an additional review, and the final selection was approved by discussion.

During data extraction stage, two authors independently reviewed the full text and tabulated the extracted data. The third reviewer extracted the data of articles that were the subject of disagreement of the first two reviewers. Extracted data included author, year of publication, HIA definition and method that was used, operationalization of walkability (if any), which NCDs were considered in HIA, aim, setting, study population of the project, policy or program, dependent and independent variables, measuring instruments, statistical/analytical methods applied, and results. In addition, conductive conditions and resources, barriers and challenges, and recommendations were extracted, if mentioned. Regarding the HIA itself, we extracted, if mentioned, who initiated the HIA, who conducted the HIA, other actors involved in the HIA, and how HIA was integrated into existing planning instruments or processes. The extracted data were captured in an Excel-Sheet that is available as supplemental material.

We conducted a qualitative analysis and summary of the extracted data to answer our research questions. The development of categories and classification of study reports was done following the principles of inductive thematic analysis [31, 32], and was done in consensus of three authors (JW, JB, EN).

## Results

### Identification and selection of relevant studies

**Fig. 2:**
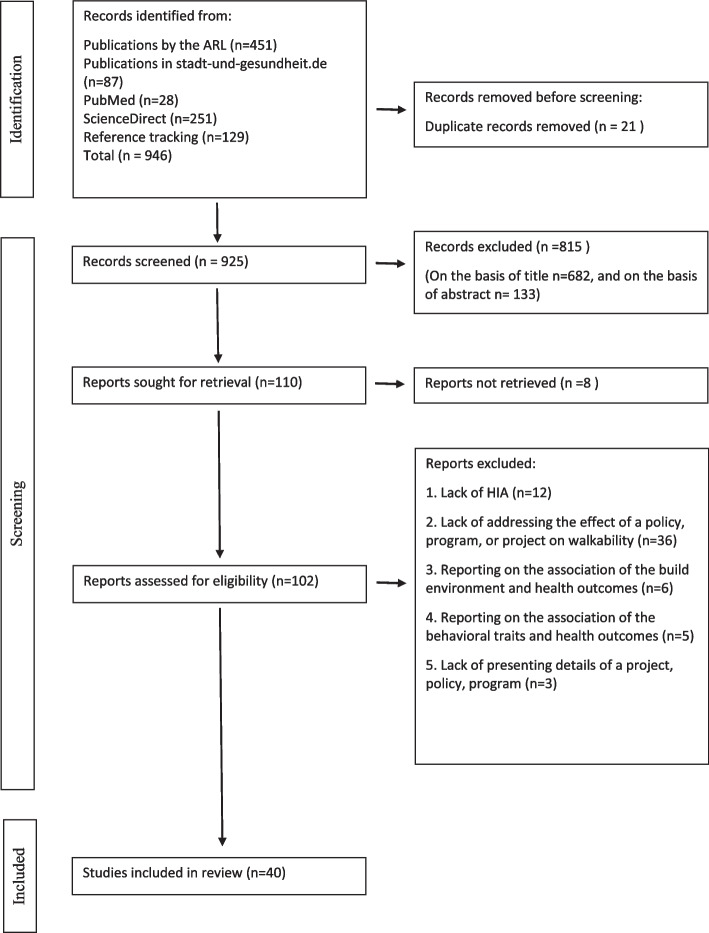
Prisma flow chart of study identification and selection

As shown in Fig. 2, a total of 946 records was identified. Database search resulted in 817 records. Additional 129 records were identified through reference tracking. 21 duplicate records were removed before title and abstract screening. Title screening excluded 682 records; abstract screening excluded additional 133 records. Since 8 reports could not be retrieved, the full text of 102 reports were assessed according to inclusion and exclusion criteria. 62 reports were excluded, most often because they did not address the effect of a policy, programme, or project on walkability (see Fig. 2). Finally, 40 study reports were included in the present review.

An overview of the included HIA reports is presented in Table 3. The complete data extraction sheet is available as supplementary material (Additional File 2).

**Table 3:** Source, place, aim of project, main results of included HIAs

Author(s), Year Place of Project	Aim of Project	Results
Agarwal et al. 2021 [33] Patna, India	The study quantifies the health benefits (reduction in mortalities) of a bicycle superhighway (BSH) in Patna, India	1. A bicycle superhighway increases the share of bicycles from 32.3% to 48.7% 2. A significant rise in the number of cyclists which is a result of better bicycle infrastructure 3. An increase in longer bicycle trips for higher-income groups 4. For the whole population, the average cycling duration has increased by 48% as a result of bicycle superhighway 5. 755 lives per year can be saved or 1640 deaths prevented/ million person as the result of increase duration of cycling 6. The monetized benefits turn out to 12.25 billion Indian rupee of saving by preventing 755 deaths annually
Andersen et al. 2017 [34] Haraldsgade district, Copenhagen, Denmark	A multicomponent urban renewal project of approximately 35 million Euros including renovation of public housing and courtyards; adding streetlights; renovation or establishment of new urban green spaces, play grounds, and sport facilities; and opening of two civic centers offering social gatherings and sport activities	Adolescents spent more time in the district and in PA in the district in 2012, after the urban renewal project
Badland et al. 2017 [35] Metropolitan Melbourne, Australia	1) Identifying spatial walking-related urban planning policies used in selected Australian states and territories; 2) creating spatial measures based on these policies; and 3) examining which, if any, are associated with transport walking in an urban context	Dwelling density and daily destinations predict walking trips Association becomes stronger for larger neighbourhood areas: walking is influenced by attributes outside the immediate neighbourhood
Bias and Abildso 2017 [36] Fairmont, West Virginia, USA	Aim of project: Creating a comprehensive bicycle and pedestrian “connectivity plan” Aim of HIA: The original HIA intended to capture broad feedback from the public around barriers to connectivity and physical activity including physical environment, safety, crime, etc Aim of study: This study evaluates policy outcomes and other effects related to HIA after 21 months of the adoption of the HIA and Connectivity Plan	1. Seven of the eleven specific recommendations can be tracked to specific outcomes in just over a year and a half after adoption of the HIA report in Fairmont 2. The city is considering the creation of a Pedestrian Safety Board to further investigate recommendations around safe active commuting 3. There are tentative plans to use the connectivity plan and HIA as the basis of an application for TIGER (Transportation Investment Generating Economic Recovery) funding
Branas et al. 2011 [37] Philadelphia, Pennsylvania, USA	A program to clean, green, and maintain abandoned vacant lots in Philadelphia, Pennsylvania. This program involved removing trash and debris, grading the land, planting grass and trees to create a park-like setting, and installing low wooden post-and-rail fences	1. Vacant lot greening was associated with consistent reductions in gun assaults and consistent reductions in vandalism 2. Vacant lot greening was associated with residents’ reporting less stress and more exercise in select sections of the city
Buekers et al. 2015 [38] Flanders, Belgium	New bicycle highways Antwerp–Mechelen and Leuven–Brussels, which were built near important traffic axes to provide the densely populated region with an alternative to car use	1. Increased PA outweighed air polution and traffic incidents 2. The benefit:cost ratio was mainly positive
Buregeya et al. 2020 [39] Quebec, Canada	Analyzing a HIA tool’s impact on the revitalization of road infrastructure, parks and green spaces, and residential housing	HIA acted in synergy with other policies or plans at the local level to foster actions favourable to health. For instance, the city’s active travel plan supported the HIA recommendations for additional cycling infrastructure. Furthermore, technical employees and elected local officials who advanced the inclusion of these recommendations in general gained a certain understanding of how transformed built environments contribute to liveable cities and a sustainable future
Chapman et al. 2018 [40] New Plymouth and Hastings, New Zealand	New Zealand’s Model Communities Programme funded cycle paths, other walking and cycling facilities, cycle parking, ‘shared spaces’, media campaigns and events, such as ‘Share the Road’, and cycle-skills training	1. Annual benefits for health in the intervention cities were estimated at 34.4 disability-adjusted life years (DALYs) and two lives saved 2. The estimated benefit/cost ratio was 11:1 and $151.2 million
Coulson et al. 2011 [41] Dings neighbourhood, Bristol, south-west England, UK	A ‘retro-fit’ model was applied, where pre-existing, residential streets were converted, and new features added. Also, a disused railway bed in neighbourhood was turned to a site for a short extension of the National Cycle Network	Several aspects of the neighbourhood were perceived to have improved, including spatial aesthetics, lighting and streetscape planting. However, influence on physical activity was minimal due to safety related concerns, poor public transport provision, local residents’ parking behaviour
Frank et al. 2019 [42] Vancouver, Canada	The Comox Greenway is a major active transportation corridor aiming to improve conditions for bicyclists of all ages and abilities	1. Participants near the greenway doubled their odds of achieving 20 min daily MVPA 2. The odds of being sedentary for more than 9 h halved for nearby residents 3. Physical activity benefits declined with increasing 100 to 500 m distance from the greenway
Frank et al. 2022 [43] San Diego county, California, USA	Palomar Gateway District redevelopment plan calls for increased housing with an emphasis on multi-family housing; additional non-residential development with a more compact urban form (i.e. multi-story buildings with less surface parking) than current development; upgrading the ridership capacity and accessibility of the light rail service along with other local bus improvements; and expansion of current pedestrian and bicycle infrastructure	1. Project increases physical activity from walking for transportation, park visitation, and reductions in type 2 diabetes and high blood pressure 2. Potential for increased exposure to air pollution among children and teens 3. The implementation of project is associated with a 9.6 percent reduction in type 2 diabetes and a 15.4 percent reduction in high blood pressure, better BMI, and general health status 4. Transportation walking increased by 67.9% for adults and 17.5% for children and teens 5. Increases in leisure walking, other leisure physical activity, moderate/vigorous PA; decreases in private automobile use, although these improvements were moderate (less than 10%) 6. Higher park visitation for all ages 7. The only health outcomes that worsened were asthma (a 10.9% increase among children and a 17.8% increase among teens) and the pedestrian/bicycle risk index (2.0% increase in risk)
Goodman et al. 2014 [44] Cardiff, Kenilworth, Southampton,UK	Connect2 initiative was established with the intention of building or improving walking and cycling routes at 79 sites across the United Kingdom	1. Living nearer the infrastructure did not predict changes in activity levels at 1-year follow-up but did predict increases in activity at 2 years relative to those living farther away (15.3 additional minutes/week walking and cycling per km nearer; 12.5 additional minutes/week of total physical activity) 2. The effects were larger among participants without car 3. Individuals living near the infrastructure did not compensate for their increased walking and cycling by reducing their participation in other types of physical activity
Gotschi 2011 [45] Portland, Oregon, USA	To assess how costs of Portland’s past and planned investments in bicycling relate to health and other benefits (cost/benefit analysis)	Benefit–cost ratio for health care and fuel saving: 3.8—.13 Benefit–cost ratio for value of statistical lifes: 53.3—20.2 Benefit–cost ratio decreasesfor plans with higher investments
Gu et al. 2017 [46] New York, USA	1. To evaluate the cost effectiveness of investments in bike lanes using New York City’s (NYC) fiscal year 2015 investment 2. To provide a generalizable model, so that localities can estimate their return on bike lane investments	1. 45.5 miles of bike lanes NYC constructed in 2015 at a cost of $8 109 511.47 may increase the probability of riding bikes by 9.32% 2. The incremental cost-effectiveness ratio (ICER) was $1297/QALY gained (is considered very cost-effective)
Guo and Gandavarapu 2010 [47] Dane County, Wisconsin, USA	Helping public investment decision makers see the greatest return on their built environment investments by developing an analysis framework for identifying the most promising improvement strategies and assessing the attainable return on investment	1. An investment of $450 million to make sidewalks available to all Dane County residents was estimated to yield a cost–benefit ratio of 1.87 over a 10-year life cycle 2. Workers were likely to drive less and walk/bike more with increasing retail accessibility 3. People who lived in neighborhoods with a higher percentage of high-income households drove more and walked less 4. Land use mix measured within 1 mile of one’s residence was associated with decreased distance walked/biked 5. Increased length of bike lane within 1/4 mile of an individual’s residence has a significantly positive impact on non-motorized travel
Hoehner et al. 2012 [48] St.Louis, Missouri, USA	Page Avenue project was a redevelopment plan including: 1. Building a new grocery store, 2. Commercial and mixed-income residential redevelopment, 3. Infrastructure improvements	1. Interdisciplinary teams are valuable but they require flexibility and organization 2. Engaging community stakeholders and decision-makers prior to, during, and following the HIA is critical to a successful HIA 3. HIA teams should not be too closely affiliated with decision-makers
Kaczynski and Sharratt 2010 [49] Williamsburg, Southwestern Ontario, Canada	Qualitative analysis of perception of residents in a newly developed neighbourhood that was designed to increase walkability	Land use diversity, safety, parks, aesthetics, sense of community are mentioned as facilitating factors for walking, lack of (old trees) is mentioned as a problem
King et al. 2010 [50] City in Colorado, USA	Discussing the benefits and challenges of applying RE-AIM to evaluate built environment strategies and recommended modest adaptations to the model; afterwards applying the revised model to 2 prototypical built environment strategies aimed at promoting healthful eating and active living	The 5 RE-AIM dimensions, with some modification of definitions, seem to be applicable to built environment interventions and provide added value given their usefulness in anticipating impact, planning for sustainability, and addressing unexpected or adverse consequences
Knuiman et al. 2014 [51] Perth, Australia	Studying the influence of built environment characteristics (walkability) and its changes over time on transport walking	1. Neighborhood walkability (especially land-use mix and street connectivity), local access to public transit stops, and variety in the types of local destinations are important determinants of walking for transportation 2. Land-use mix had a greater and more significant relationship than did either street connectivity or residential density with transport walking
MacDonald et al. 2010 [52] Charlotte NC, US	To examine the cross-sectional associations between objective and perceived measures of the built environment; BMI; obesity pre- and post-LRT (light rail transit) construction	1. More-positive perceptions of one’s neighborhood at baseline were associated with lower BMI; 15% lower odds of obesity; 9% higher odds of meeting weekly RPA through walking; and 11% higher odds of meeting RPA levels of vigorous exercise 2. The use of LRT to commute to work was associated with an average 1.18 reduction in BMI and 81% reduced odds of becoming obese over time
MacDonald Gibson et al. 2015 [53] Raleigh, North Carolina, USA	Developing a computer simulation model for forecasting the health effects of urban features that promote walking	The simulation model predicts that the plan would increase average daily time spent walking for transportation by 17 min. As a result, annual deaths from all causes are predicted to decrease by 5.5%. Annual new cases of diabetes, coronary heart disease, stroke, and hypertension are predicted to decline by 1.9%, 2.3%, 1.3%, and 1.6%, respectively. The present value of these health benefits is $21,000 per resident
Mansfield and Gibson 2016 [54] City Neighbourhoods in metropolitan areas, USA	Developing statistical models to estimate health impacts of alternative city planning scenarios (with and without infrastructure) to support active transportation (walking and cycling), and reduction of premature death without any referral to a specific disease. Application of mathematical models in hypothetical HIAs	1. Increase in the number of walking and biking trips .2. Increased population density and percentage of rental units increased bike trips 3. Overall increase in duration of walking and biking trips 4. Built environment variables have small but significant effects on daily walking time but no significant effects on daily biking time 5. Case study of Raleigh–Durham–Chapel Hill: 5.a. The statistical model was able to predict observed transportation physical activity in the Raleigh–Durham–Chapel Hill region to within 0.5 MET-hours per day (equivalent to about 9 min of daily walking time) for 83% of observations 5.b. Across the Raleigh–Durham–Chapel Hill region, estimated 38 (95% CI 15–59) premature deaths potentially could be avoided if the entire population walked 37.4 min per week for transportation 5.c. If changes to the built environment induced 14.5% of drivers to commute by public transit, estimated 6.2 (95% CI 2.6–10.3) premature deaths could have been prevented in 2013
Mansfield and Gibson 2015 [55] North Carolina, USA	To demonstrate the use of DYNAMO-HIA for supporting health impact assessments of transportation infrastructure projects	1. In the BRRC, DYNAMO-HIA estimates a significant reduction in premature all-cause mortality as well as significant preventive effects for hypertension, type 2 diabetes mellitus, and CHD 2. In Sparta, significant reductions in premature mortality, cases of hypertension, and cases of type 2 diabetes mellitus are estimated; however, estimated effects on avoided cases of CHD are minimal 3. In Winterville, DYNAMO-HIA estimates small, yet significant, reductions in premature mortality and cases of hypertension and minimal effects on type 2 diabetes and CHD 4. Across all sites, no significant reductions in cases of stroke are estimated
Mansfield et al. 2015 [56] Raleigh-Durham-Chapel Hill, North Carolina, USA	Modelling impact of 3 scenarios (base case, compact growth and increased sprawl) of urban development on air quality and mortality	1. Compact development slightly decreases (-0.2%) point estimates of regional annual average PM2.5 concentrations, while sprawling development slightly increases (+ 1%) concentrations 2. Point estimates of health impacts are in opposite directions: compact development increases (+ 39%) and sprawling development decreases (-33%) PM2.5 -attributable mortality 3. Compactness increases local variation in PM2.5 concentrations and increases the severity of local air pollution hotspots
Mueller et al. 2018 [57] Spain, UK, Belgium, Austria, Germany, Switzerland, Italy	Estimating the impact of cycling network expansions in seven European cities	1. A cycling network of 315 km/100,000 persons lead to cycling mode share of 24.7% 2. A cycling mode share of 24.7% could prevent 10,000 premature deaths 3. Benefits of increases in PA outweighed detriments of air pollution and traffic incidents 4. Air pollution exposure have higher risk than fatal traffic incident 5. Senario1 (10% expansion of cycling network) had the largest cost–benefit ratios 6. S4 (expansion of all streets) produced greatest benefits among other scenarios with high increase of cycling and lower annual premature deaths
Mueller et al. 2020 [58] Barcelona, Spain	Implementation of the Superblock Model in Barcelona/Spain. Superblocks are blocks of streets with pacified interior streets that are devoted to active transport and residential traffic	1. Prevention of 667 annual premature deaths 2. Greatest number of preventable deaths could be attributed to reductions in NO2, followed by noise, heat, and green space development 3. Increased PA for an estimated 65,000 persons shifting car/motorcycle trips to public and active transport resulted in 36 preventable deaths 4. An average increase in life expectancy for the adult population of almost 200 days 5. Annual economic impact of 1.7 billion EUR
Nicholas et al. 2019 [59] Los Angeles, California, USA	1. To determine the health impacts of three future scenarios of travel behavior by mode 2. To provide specific recommendations for how to conduct health impact assessments of local transportation plans	1. The largest impacts were on cardiovascular disease through increases in physical activity 2. Reductions in air pollution–related illnesses were more modest 3. Traffic injuries and deaths increased across all scenarios but were greatly reduced through targeted roadway safety enhancements 4. Both aspirational scenarios produce net savings ($79 million and $162 million, respectively), the conservative scenario produces net costs attributable to the high costs of traffic injuries
Panter et al. 2016 [60] Cambridge, UK	The Cambridgeshire Guided Busway comprised a new bus network and an adjacent 22-km traffic-free walking and cycling route in and around Cambridge	1. Exposure to the busway was associated with a significantly greater likelihood of an increase in weekly cycle commuting time 2. An increase in overall time spent in active commuting among the least active commuters at baseline
Payton Foh et al. 2021 [61] Newark, New Jersey, USA	Opening of park for recreational activities	1. Self-reported neighborhood walkability was associated with increased walking (*P* = .01) 2. Increased perception of neighborhood safety was associated with less walking (*P* = .01) 3. Positive changes associated with improvements to the built environment may be limited by social conditions such as neighborhood violence 4. Physical activity policies or interventions aimed at increasing access to open spaces must involve comprehensive, multi-pronged approaches that recognize the realities of the local social context to ensure their long-term success
Perdue et al. 2012 [62] Oregon, USA	To inform the debate, within a state legislature, about the value of state policy and provide information for local planning agencies to better incorporate health considerations into planning activities	1. Increasing the cost of driving was not consistently found to reduce air pollution, increase physical activity, or reduce collisions 2. Strengthening public transit was associated with increased levels of physical activity 3. Altering the built environment was associated with increased physical activity and decreased air pollution
Ross et al. 2012 [63] Atlanta, Georgia, USA	The BeltLine project will transform a 22-mile loop of an abandoned railroad and surrounding property to 2100 acres of parks; 33 miles trails; 22 miles of transit, 6500 acres of redevelopment, 30,000 new jobs, plus sidewalk, streetscape, road, and intersection improvements	1. Giving priority to the construction of trails and greenspace rather than residential and retail construction, 2. Making health an explicit goal in project, 3. Adding a public health professional to decision-making boards, 4. Increasing the connectivity between the BeltLine and civic spaces, 5. Ensuring affordable housing
Stevenson et al. 2016 [64] Melbourne, Australia; London UK; Boston, USA; Copenhagen, Denmark; Sao Paulo, Brasil; Delhi, India;	Estimation of the population health effects arising from alternative land-use and transport policy initiatives (modelling compact city scenarios) in six cities using a health impact assessment framework	1. Modelled compact city scenario resulted in health gains for all cities (for diabetes, cardiovascular disease, and respiratory disease) with overall health gains of 420–826 disability-adjusted life-years (DALYs) per 100 000 population due to modal shift towards walking and cycling 2. For moderate to highly motorised cities, such as Melbourne, London, and Boston, the compact city scenario predicted a small increase in road trauma for cyclists and pedestrians (health loss of between 34 and 41 DALYs per 100 000 population)
Thornton et al. 2013 [65] Baltimore, Maryland, USA	Health impact assessment (HIA) of a rezoning effort in Baltimore (TransForm Baltimore) by highlighting its' effects on multiple health outcomes, including physical activity, violent crime, and obesity	1. Health was not an active goal for TransForm Baltimore for many city offcials and expert consultants 2. Mixed-use developments are associated with increasing physical activity, decreasing obesity and obesity-related illness especially for socioeconomically advantaged populations 3. Increased mixed-use developments can be associated with increased crime 4. Higher density of alcohol sales outlets is associated with an increased risk of violent crime 5. TransForm Baltimore would increase the percentage of city residents living in neighborhoods with pedestrian-oriented design from 1 to 24% 6. The TransForm Baltimore HIA identified mixed-use development as an important mechanism for impacting health through zoning via potential impacts on physical activity, violent crime, and obesity
Tiwari et al. 2016 [66] 3 Cities in India	Assess impact of improving non-motorized traffic infrastructure and public transport infrastructure on CO2 emissions and traffic fatalities	1. Maximum reduction in CO2 emissions and highest improvement in safety and reduce traffic fatalities is achieved when both PT (Public transport) and NMT (non-motorized transport) infrastructure are improved 2. Improving only PT infrastructure may have marginal effect on overall reduction of CO2 emissions and adverse effects on traffic safety 3. NMT infrastructure is crucial for maintaining the travel mode shares in favor of PT and NMT in future
Tully et al. 2013 [67] Belfast, Northern Ireland, UK	Connswater Community Greenway: major inner city urban regeneration project: The project involves: 1. A major urban regeneration project called The Connswater Community Greenway in Belfast including a. Development of a 9 km linear park, and new cycle paths and walkways b. Improvement of the aesthetics of shared public spaces (i.e. planting trees/shrubs, erection of public art) c. Remediation of water courses to improve the natural diversity and reduce the risk of flooding d. Change perception of safety in the community through 24/7 lighting, CCTV and the presence of park wardens 2. A number of programmes to promote physical activity in the area (i.e. extension of neighbourhood walking groups, schools-based initiatives, community-based social marketing initiatives)	This article is a protocol, therefore there are no outcomes
Veerman et al. 2016 [68] Perth, Australia	Analysing the cost-effectiveness of extending the length of sidewalks in a neighbourhood to increase levels of walking and improve health	1. Investing in the length of sidewalks is unlikely to be a cost-effective method of improving health at the existing levels of residential density in Perth 2. 10 km of sidewalk in an average neighbourhood with 19,000 adult residents was estimated to cost A$4.2 million over 30 years and gain 24 HALYs over the lifetime of an average neighbourhood adult resident population 3. The incremental cost-effectiveness ratio was A$176 000/HALY 4. Increasing population densities improves cost-effectiveness by spreading the fixed costs of neighbourhood improvements over more people, and leading to greater overall benefit, which improves cost effectiveness
Woodcock et al.2013 [69] England and Wales (London was excluded), UK	Evaluation of health and environmental impacts of high walking and cycling transport scenarios	1. Considerable reductions in disease burden 2. The largest health gains were from changes in ischemic heart disease, stroke, and dementia, followed by reductions in injuries 3. Largest benefits resulted from,in order, physical activity, road traffic injuries, and air pollution
Zapata-Diomedi et al. 2016 [70] Brisbane, Perth and Adelaide, Australia	Deriving scenarios from the literature for the association between built environment attributes and physical activity, then using a mathematical model to translate improvements in physical activity to health-adjusted life years (HALYs) and healthcare costs	1. Density had no statistically significant estimation with gained HALYs 2. No significant result on the impact of diversity on physical activity/HALYs 3. Design has increasing impact on HALYs via connectivity (intersec-tions within area), availability of side-walks within neigh-bourhood, street lights 4. Providing additional recreational destination increases HALYs 5. Availability of bus stops increases walking for transport, gains HALYs 6. Improvements in walking for transport (measured in standardised walkability index) have positive impact on HALYs 7. Health care cost savings due to prevented physical activity-related diseases ranged between A$1300 to A$105,355 per 100,000 adults per year 8. Additional health care costs of prolonged life years attributable to improvements in physical activity were nearly 50% higher than the estimated health care costs savings
Zapata-Diomedi et al. 2019 [71] Melbourn, Australia	Plan Melbourne aims to influence housing supply within the existing urban area by industrial land redevelopment and new suburbs on the city’s fringe Plan Melbourne addresses: 1. transport congestion 2. employment 3. public transport 4. services accessibility 5. housing affordability 6. environmental sustainability	1. Altona North Developed vs Truganina: a. greater housing density (9 vs 3.5 dwellings per hectare), b. greater diversity of land uses (0.74 vs 0.53), c. destination accessibility train station, bus stop and supermarket, and 11 local living destination score, while, Truganina only had access to bus stops, and 8 local living destinations score d. higher Design number of intersections (54 vs 33 /sq.km), e. higher probability of transport walking (48% vs 26%), f. an Altona North Developed resident was estimated to walk 131 min per week for transport purposes, while a Truganina resident was estimated to walk 71 min per week 2. The new development in Altona North is not changing the destination mix and density significantly 3. There will be modest improvements in the probability of walking i.e.only 2% as the result of new develpment in Altona North 4. If the population (21,000) of greenfield neighbourhood (similar to Truganina) were exposed to the urban development form in a brownfield neighbourhood (similar to Altona North Developed) the decrease in incidence and mortality of physical inactivity-related chronic diseases would lead to 1600 HALYs and economic benefits of A$94 million 5. Well-located higher density brownfield developments in established areas with existing amenities are likely to produce better health outcomes and economic benefits compared with continuing to house people in low density developments on the urban fringe
Zapata-Diomedi et al. 2018 [72] Australia	Calculating monetised PA-related health benefits of walking and cycling	Results indicate that the value of PA-related health benefits associated with walking is A$0.98 (95% Uncertainty Interval (UI) 0.73 to 1.24) per kilometre. For cycling the benefits are worth A$0.62 (95% UI 0.46 to 0.79) per kilometre

### Location, type of projects, and type of HIAs

Most of the identified published HIAs addressed projects or policies in the USA (*n* = 18), followed by Australia (*n* = 6), UK (*n* = 5), and Canada (*n* = 3). Moreover, there were two reports from India, one from New Zealand, one that covered several European countries, one that covered several European and Non-European Countries, and another three reports each covering one European country. Particularly, Germany was only addressed in one HIA as one of several European countries (see Additional file 1: supplementary table S1 for details).

Most of the HIAs investigated the impact of improving or extending the infrastructure to facilitate active transport or public transport (*n* = 13). This refers to more concrete projects like extending cycling networks or better sidewalks. Among them, five HIAs were about improving bicycle and pedestrian infrastructure, and another five addressed bicycle infrastructure alone. Respectively one HIA studied improving public transport infrastructure alone, non-motorized transport plus public transport infrastructure, or extending sidewalks. Additional 11 HIAs assessed the impact of more general scenarios or policies to support active transport (see Additional file 1: suppl. table S2 for details).

Six HIAs examined the development of new suburbs; the redevelopment, revitalisation, or regeneration of a city or abandoned areas: Another five HIAs examined the redesign of urban neighbourhoods.

33 HIAs were clearly quantitative HIAs, that aimed at quantifying at least one primary health outcome, four HIAs were clearly qualitative HIAs and three reports included quantitative and qualitative reports (see Additional file 1: suppl. table S3 for details).

### Data sources and analysis

A variety of data sources was used as basis for HIAs, this included primary data collection, secondary use of existing data, measurement of built environment variables via geographic information systems (GIS), and analysis of existing reports, inventories, or other similar data (see Addirional file 1: suppl. table S5 for details). Primary data collection through surveys or questionnaires was used in eight HIAs, interviews and/or focus groups and group discussions in seven HIAs, structured observations and audits in three HIAs, and accelerometer measurement in one HIA.

The secondary use of existing surveys was applied in 14 HIAs. The secondary use of data from group discussions, interviews and travel diaries was each mentioned in one HIA respectively.

Literature reviews as basic data source were used in five HIAs, the measurement of built environment variables by GIS was applied in seven HIAs, and the analysis of existing reports, inventories or other types of data was applied in seven HIAs.

### Health impacts

Cardiovascular diseases were the health endpoint that was investigated most often, in 16 HIAs. This was followed by diabetes in 12 HIAs, Cancer (8 HIAs), mental illness (6 HIAs), premature death (5 HIAs), all-cause mortality (5 HIAs), respiratory diseases (5 HIAs), traffic accidents (4 HIAs) and obesity (4 HIAs) (see Additional file 1: suppl. table S4).

Most of the HIAs (*n* = 31) reported the improvement of health or health behaviour resulting from the investigated project or policy. However, three HIAs reported a lack of improvement or even a decrease of health status. In parallel, 13 HIAs reported a gain in economic value, whereas one reported a lack or loss of economic effects. Moreover, three HIAs reported on social effects and six HIAs gave additional recommendations for policies or the implementation of projects or HIAs (see Additional file 1: suppl. table S6 for details).

A closer look at those HIAs that reported negative health impacts or failed to find a positive impact yields the following results. One HIA that reported negative health impacts was on a comprehensive transit-oriented district redevelopment plan, that would result in increased exposure to air pollution and increased rate of asthma in children on one hand, but increased physical activity from walking and reductions in type 2 diabetes and high blood pressure on the other hand [43]. A HIA of different scenarios of urban development on air quality concluded that compact development increases particulate matter PM2.5 concentrations and PM2.5 attributable mortality [55]. Finally, a qualitative HIA using focus groups of a neighbourhood transformation project failed to find noticeable increases of physical activity [41].

Among the social effects reported, there was the potential for more alcohol outlets in mixed-use developments of a rezoning project in Baltimore, and associated increasing violent crime [65]. In contrast, greening of a vacant urban space was associated with reductions in gun assaults, vandalism and stress, and more safety [37]. Finally, in a newly developed neighbourhood that was designed to improve walkability, residents reported safety, aesthetics, and sense of community as factors that facilitated walking [49].

### Implementation of HIAs

Regarding the implementation of the HIAs, none of the reports mentioned explicitly who was doing the work, but it reasonable to assume that this was done by the listed authors.

Most of the studies (32 of 40) reported external funding of the HIA and few studies explicitly mentioned that the HIA was initiated by a particular institution.

Only two study reports mentioned other institutions or stakeholders that were actively involved in the HIA: the city where the project was located, the respective Metro company and the NIH in one study, a number of local organizations, the police department, the church priest and local farmers and ranchers in the other study.

None of the study reports mentioned whether or how the HIA was integrated or associated with other planning instruments or procedures.

## Discussion

The present review aims at summarizing the peer-reviewed literature on health impact assessments of walkability in urban development. The vast majority of studies reported beneficial health impacts particularly reductions of non-communicable diseases like cardiovascular diseases, diabetes, cancer, and mortality. As most HIAs examined the impact of projects, plans or scenarios that aimed at increasing active transport and/or public transport these results are in line with prior findings, because the beneficial consequences of active transport, walking and cycling have been well established [14, 20]. Negative health impacts were only reported in two HIAs and were related to increased exposure to air pollution that may result from more walking and cycling [43, 55]. However, it has been shown that beneficial impacts of walking and cycling clearly outweigh the potential negative impacts from air pollution and traffic accidents.

Walkability in a wider sense was studied in eleven HIAs, by either investigating the redevelopment of cities or abandoned areas, the development of new suburbs or the redesign of urban neighbourhoods. With one exception these HIAs reported beneficial health impacts. Social effects were rarely addressed in the included HIAs and differed fundamentally among the examined projects or plans. Mixed-use neighbourhoods could result in more alcohol outlets and more violent crime [65], whereas greening of vacant urban space could result in reduced violence and vandalism and increased perceived safety of the residents [37]. A newly developed neighbourhood designed to improve walkability was associated with improved perceived safety, aesthetics and sense of community [49].

In summary, the present review clearly shows that improving the walkability of neighbourhoods or cities yields positive health impacts and has only limited negative effects, depending on the project or policy. Moreover, improving infrastructure and opportunities for active transport and public transport play a major role for the beneficial effects. However, the research reports included in this review did not address aspects of inequality and equity, i.e. whether beneficial or harmful effects of projects, policies or programmes are equally distributed among different population groups. There is a need for future research to address the social inequalities of health impacts as well. Otherwise, it could happen that walkability is primarily improved in more affluent neighbourhoods, whereas more deprived neighbourhoods are even more neglected.

This review has some limitations that have to be considered when drawing conclusions. Most importantly, due to our search and selection strategy we may have missed several relevant HIA reports. First, we limited our search on peer-reviewed papers that were identified through two databases and two websites of German organizations and follow-up reference tracking. Thus, HIA reports in the grey literature were not included. It is likely that several HIA reports exist either unpublished or available only on local or regional websites. Indeed, this has been confirmed by a UK expert in health impact assessment (personal communication Dr. Fischer, Liverpool).

Secondly, we explicitly included walkability in our search terms which may cause restriction of the identified results. For example, the article by Mueller et al. [58] examines the health impacts of the superblock design in Barcelona, Spain. The superblock design is clearly improving the walkability of neighbourhood blocks as it “… aims to reclaim space for people, reduce motorized transport, promote sustainable mobility and active lifestyles, provide urban greening …” [58]. However, the whole article does not even mention the term “walkability”. Nevertheless, we hope that we have covered the most important HIAs related to walkability through our reference tracking which identified the mentioned article on the Barcelona superblocks.

Having the limitations in mind, the following conclusions seem justified. Health impact assessments related to the walkability of urban environments are more established in English speaking countries, including US, UK, Canada, Australia, and New Zealand. Other European countries, particularly Germany, clearly lack behind. Despite the 40 HIA reports that were included in our review, there is a need for more HIAs published in peer-reviewed journals, given the important role urban environments play in determining human health. Peer-reviewed publication would provide some basic quality assurance and hence increase the credibility of the findings, and in addition would help to identify relevant reports more easily through standard scientific literature data bases. This in turn could motivate more research on the health impacts of walkability and underline the importance of considering and improving walkability in urban planning processes. If not considerably more unpublished HIAs exist, we have to conclude that health impacts of urban planning and urban development need a considerable push in local administrations and planning agencies.

A major strength of this review is going beyond active and public transport; it also includes more general characteristics of the urban built environment which are subsumed under the concept of walkability.

## Conclusions

While the beneficial effects of active transport and public transport which are important components of walkability are well documented and established, the contribution of other features or components of walkability to health are less well understood. Particularly, there is a need to establish more quantitative associations of the different dimensions of walkability as suggested in the walkability framework [27] to increased physical activity, social interaction and perceived safety and stress. Such quantitative associations would allow to predict the health impact of urban design features more accurately.

Future research would benefit from explicitly mentioning the key terms like “health impact” or “walkability” in the title of their publications, and from providing more clear definitions of such constructs in their report. With regard to public health practice, reports should provide more detail about who initiated HIAs, who conducted them, who participated and how they were implemented in the planning processes. Finally, it is desirable, that access to existing HIAs is facilitated by publishing them in peer-review, preferably open access scientific journals. This could promote the consideration of health impacts in urban planning, particularly in countries and regions were this is not well-established practice.

## Supplementary Information

**Additional file 1: Table S1.** Geographic distribution of HIAs. **Table S2.** Types of projects, programs or policies that is evaluated using HIA. **Table S3.** Type of HIA. **Table S4.** Type of health endpoints. **Table S5.** Type of data source for the HIA. **Table S6.** Type of results.

**Additional file 2: **Data extraction sheet.

## Data Availability

The data extraction sheet produced for the literature review is available as Additional file 2 (Excel File).

## References

[1] Dahlgren G, Whitehead M. Policies and strategies to promote social equity in health. 1991. Verfügbar unter: https://s2.medicina.uady.mx/observatorio/docs/eq/li/Eq_2007_Li_Dahlgren.pdf. Accessed 5 Apr 2016.

[2] World Health Organization, UN Habitat. Global report on urban health: equitable, healthier cities for sustainable development. World Health Organization; 2016. https://apps.who.int/iris/handle/10665/204715. Accessed 15 Jul 2019.

[3] Fehr R, Viliani F, Nowacki J, Martuzzi M (2014). Health in Impact Assessments: Opportunities not to be missed.

[4] WHO Regional Office for Europe, European Centre for Health Policy. Health Impact Assessment. Main concepts and suggested appraoch. 1999. http://www.impactsante.ch/pdf/HIA_Gothenburg_consensus_paper_1999. Accessed 25 Mar 2019.

[5] Remoundou K, Koundouri P (2009). Environmental Effects on Public Health: An Economic Perspective. IJERPH.

[6] World Health Organization. Health Impact Assessment Toolkit for Cities. 2005. https://www.euro.who.int/__data/assets/pdf_file/0007/101500/HIA_Toolkit_1.pdf. Accessed 17 Jan 2022.

[7] World Health Organization. The Helsinki Statement on Health in All Policies. 2013. https://www.who.int/healthpromotion/conferences/8gchp/8gchp_helsinki_statement.pdf. Accessed 17 Feb 2022

[8] World Health Organization. Health impact assessments. https://www.who.int/tools/health-impact-assessments. Accessed 17 Jan 2022.

[9] Winkler MS, Furu P, Viliani F, Cave B, Divall M, Ramesh G (2020). Current Global Health Impact Assessment Practice. Int J Environ Res Public Health.

[10] World Health Organization (2010). Global recommendations on physical activity for health.

[11] GBD 2019 Risk Factors Collaborators (2020). Global burden of 87 risk factors in 204 countries and territories, 1990–2019: a systematic analysis for the Global Burden of Disease Study 2019. Lancet.

[12] World Health Organization. WHO guidelines on physical activity and sedentary behaviour. Geneva: World Health Organization; 2020. https://apps.who.int/iris/handle/10665/336656. Accessed 4 Feb 2022.

[13] Hruby A, Manson JE, Qi L, Malik VS, Rimm EB, Sun Q (2016). Determinants and Consequences of Obesity. Am J Public Health.

[14] Kahlmeier S, World Health Organization, Regional Office for Europe (2013). Health economic assessment tools (HEAT) for walking and for cycling: methodology and user guide : economic assessment of transport infrastructure and policies.

[15] Kelly P, Kahlmeier S, Götschi T, Orsini N, Richards J, Roberts N (2014). Systematic review and meta-analysis of reduction in all-cause mortality from walking and cycling and shape of dose response relationship. Int J Behav Nutr Phys Act.

[16] Wen CP, Wai JPM, Tsai MK, Yang YC, Cheng TYD, Lee MC (2011). Minimum amount of physical activity for reduced mortality and extended life expectancy: a prospective cohort study. Lancet.

[17] Kyu HH, Bachman VF, Alexander LT, Mumford JE, Afshin A, Estep K (2016). Physical activity and risk of breast cancer, colon cancer, diabetes, ischemic heart disease, and ischemic stroke events: systematic review and dose-response meta-analysis for the Global Burden of Disease Study 2013. BMJ.

[18] Sieverdes JC, Ray BM, Sui X, Lee DC, Hand GA, Baruth M (2012). Association between leisure time physical activity and depressive symptoms in men. Med Sci Sports Exerc.

[19] Sofi F, Valecchi D, Bacci D, Abbate R, Gensini GF, Casini A (2011). Physical activity and risk of cognitive decline: a meta-analysis of prospective studies. J Intern Med.

[20] Mueller N, Rojas-Rueda D, Cole-Hunter T, de Nazelle A, Dons E, Gerike R (2015). Health impact assessment of active transportation: A systematic review. Prev Med.

[21] Saelens BE, Sallis JF, Black JB, Chen D (2003). Neighborhood-based differences in physical activity: an environment scale evaluation. Am J Public Health.

[22] Saelens BE, Sallis JF, Frank LD (2003). Environmental correlates of walking and cycling: findings from the transportation, urban design, and planning literatures. Ann Behav Med.

[23] Saelens BE, Handy SL (2008). Built environment correlates of walking: a review. Med Sci Sports Exerc Juli.

[24] Kerr J. Definitions and Dimensions of Walkability. In: Bucksch J, Schneider S, Herausgeber. Walkability: das Handbuch zur Bewegungsförderung in der Kommune. 1. Auflage. Bern: Verlag Hans Huber; 2014. S. 143–51. (Verlag Hans Huber Programmbereich Gesundheit).

[25] Bucksch J, Schneider S. Vorwort. In: Bucksch J, Schneider S, Herausgeber. Walkability: das Handbuch zur Bewegungsförderung in der Kommune. 1. Auflage. Bern: Verlag Hans Huber; 2014. S. 9–11. (Verlag Hans Huber Programmbereich Gesundheit).

[26] Zuniga-Teran AA. From Neighborhoods to Wellbeing and Conservation: Enhancing the use of Greenspace Through Walkability [Internet]. University of Arizona; 2015. https://repository.arizona.edu/bitstream/handle/10150/555990/azu_etd_13806_sip1_m.pdf?sequence=1. Accessed 11 May 2021.

[27] Zuniga-Teran AA, Orr BJ, Gimblett RH, Chalfoun NV, Marsh SE, Guertin DP (2017). Designing healthy communities: Testing the walkability model. Front Arch Res.

[28] White MP, Alcock I, Wheeler BW, Depledge MH (2013). Would you be happier living in a greener urban area? A fixed-effects analysis of panel data. Psychol Sci Juni.

[29] Hua J, Mendoza-Vasconez AS, Chrisinger BW, Conway TL, Todd M, Adams MA (2022). Associations of social cohesion and quality of life with objective and perceived built environments: a latent profile analysis among seniors. J Public Health (Oxf).

[30] Page MJ, McKenzie JE, Bossuyt PM, Boutron I, Hoffmann TC, Mulrow CD (2021). The PRISMA 2020 statement: an updated guideline for reporting systematic reviews. BMJ.

[31] Mayring P. Qualitiative Inhaltsanalyse: Grundlagen und Techniken. 12. Auflage. Weinheim, Basel: Beltz; 2015.

[32] Pope C, Ziebland S, Mays N (2000). Qualitative research in health care. Analysing qualitative data. BMJ.

[33] Agarwal A (2021). Quantifying Health & Economic Benefits of Bicycle Superhighway: Evidence from Patna. Procedia Comput Sci.

[34] Andersen HB, Christiansen LB, Klinker CD, Ersbøll AK, Troelsen J, Kerr J (2017). Increases in Use and Activity Due to Urban Renewal: Effect of a Natural Experiment. Am J Prev Med.

[35] Badland H, Mavoa S, Boulangé C, Eagleson S, Gunn L, Stewart J (2017). Identifying, creating, and testing urban planning measures for transport walking: Findings from the Australian national liveability study. JTH.

[36] Bias TK, Abildso CG (2017). Measuring policy and related effects of a health impact assessment related to connectivity. Prev Med.

[37] Branas CC, Cheney RA, MacDonald JM, Tam VW, Jackson TD, Ten Have TR (2011). A Difference-in-Differences Analysis of Health, Safety, and Greening Vacant Urban Space. Am J Epidemiol.

[38] Buekers J, Dons E, Elen B, Int Panis L (2015). Health impact model for modal shift from car use to cycling or walking in Flanders: application to two bicycle highways. J Trans Health.

[39] Buregeya JM, Loignon C, Brousselle A (2020). Contribution analysis to analyze the effects of the health impact assessment at the local level: A case of urban revitalization. Eval Program Plann.

[40] Chapman R, Keall M, Howden-Chapman P, Grams M, Witten K, Randal E (2018). A Cost Benefit Analysis of an Active Travel Intervention with Health and Carbon Emission Reduction Benefits. Int J Environ Res Public Health.

[41] Coulson JC, Fox KR, Lawlor DA, Trayers T (2011). Residents’ diverse perspectives of the impact of neighbourhood renewal on quality of life and physical activity engagement: Improvements but unresolved issues. Health Place.

[42] Frank LD, Hong A, Ngo VD (2019). Causal evaluation of urban greenway retrofit: A longitudinal study on physical activity and sedentary behavior. Prev Med.

[43] Frank LD, Fox EH, Ulmer JM, Chapman JE, Braun LM (2022). Quantifying the health benefits of transit-oriented development: Creation and application of the San Diego Public Health Assessment Model (SD-PHAM). Transport Policy.

[44] Goodman A, Sahlqvist S, Ogilvie D (2014). New Walking and Cycling Routes and Increased Physical Activity: One- and 2-Year Findings From the UK iConnect Study. Am J Public Health.

[45] Gotschi T (2011). Costs and Benefits of Bicycling Investments in Portland. Oregon. J Phys Act Health.

[46] Gu J, Mohit B, Muennig PA (2017). The cost-effectiveness of bike lanes in New York City. Inj Prev.

[47] Guo JY, Gandavarapu S (2010). An economic evaluation of health-promotive built environment changes. Prev Med.

[48] Hoehner CM, Rios J, Garmendia C, Baldwin S, Kelly CM, Knights DM (2012). Page Avenue health impact assessment: Building on diverse partnerships and evidence to promote a healthy community. Health Place.

[49] Kaczynski AT, Sharratt MT (2010). Deconstructing Williamsburg: Using focus groups to examine residents’ perceptions of the building of a walkable community. Int J Behav Nutr Phys Act.

[50] King DK, Glasgow RE, Leeman-Castillo B (2010). Reaiming RE-AIM: Using the Model to Plan, Implement, and Evaluate the Effects of Environmental Change Approaches to Enhancing Population Health. Am J Public Health.

[51] Knuiman MW, Christian HE, Divitini ML, Foster SA, Bull FC, Badland HM (2014). A Longitudinal Analysis of the Influence of the Neighborhood Built Environment on Walking for Transportation: The RESIDE Study. Am J Epidemiol.

[52] MacDonald JM, Stokes RJ, Cohen DA, Kofner A, Ridgeway GK (2010). The Effect of Light Rail Transit on Body Mass Index and Physical Activity. Am J Prev Med.

[53] MacDonald Gibson J, Rodriguez D, Dennerlein T, Mead J, Hasch T, Meacci G (2015). Predicting urban design effects on physical activity and public health: A case study. Health Place.

[54] Mansfield TJ, Gibson JM (2016). Estimating Active Transportation Behaviors to Support Health Impact Assessment in the United States. Front Public Health.

[55] Mansfield TJ, Gibson JM (2015). Health Impacts of Increased Physical Activity from Changes in Transportation Infrastructure: Quantitative Estimates for Three Communities. BioMed Res Int.

[56] Mansfield TJ, Rodriguez DA, Huegy J, MacDonald GJ (2015). The Effects of Urban Form on Ambient Air Pollution and Public Health Risk: A Case Study in Raleigh. North Carolina Risk Analysis.

[57] Mueller N, Rojas-Rueda D, Salmon M, Martinez D, Ambros A, Brand C (2018). Health impact assessment of cycling network expansions in European cities. Prev Med.

[58] Mueller N, Rojas-Rueda D, Khreis H, Cirach M, Andrés D, Ballester J (2020). Changing the urban design of cities for health: The superblock model. Environ Int.

[59] Nicholas W, Vidyanti I, Caesar E, Maizlish N (2019). Routine Assessment of Health Impacts of Local Transportation Plans: A Case Study From the City of Los Angeles. Am J Public Health März.

[60] Panter J, Heinen E, Mackett R, Ogilvie D (2016). Impact of New Transport Infrastructure on Walking, Cycling, and Physical Activity. Am J Prev Med.

[61] Payton Foh E, Brown RR, Denzongpa K, Echeverria S (2021). Legacies of Environmental Injustice on Neighborhood Violence, Poverty and Active Living in an African American Community. Ethn Dis.

[62] Perdue LA, Michael YL, Harris C, Heller J, Livingston C, Rader M, Goff NM. Rapid health impact assessment of policies to reduce vehicle miles traveled in Oregon. Public Health. 2012;126(12):1063–71. 10.1016/j.puhe.2011.09.026. 10.1016/j.puhe.2011.09.026 22818411

[63] Ross CL, Leone de Nie K, Dannenberg AL, Beck LF, Marcus MJ, Barringer J (2012). Health Impact Assessment of the Atlanta BeltLine. Am J Prev Med.

[64] Stevenson M, Thompson J, de Sá TH, Ewing R, Mohan D, McClure R (2016). Land use, transport, and population health: estimating the health benefits of compact cities. Lancet.

[65] Thornton RLJ, Greiner A, Fichtenberg CM, Feingold BJ, Ellen JM, Jennings JM (2013). Achieving a Healthy Zoning Policy in Baltimore: Results of a Health Impact Assessment of the TransForm Baltimore Zoning Code Rewrite. Public Health Rep.

[66] Tiwari G, Jain D, Ramachandra Rao K (2016). Impact of public transport and non-motorized transport infrastructure on travel mode shares, energy, emissions and safety: Case of Indian cities. Transp Res D Transp Environ.

[67] Tully MA, Hunter RF, McAneney H, Cupples ME, Donnelly M, Ellis G (2013). Physical activity and the rejuvenation of Connswater (PARC study): protocol for a natural experiment investigating the impact of urban regeneration on public health. BMC Public Health.

[68] Veerman JL, Zapata-Diomedi B, Gunn L, McCormack GR, Cobiac LJ, Mantilla Herrera AM (2016). Cost-effectiveness of investing in sidewalks as a means of increasing physical activity: a RESIDE modelling study. BMJ Open.

[69] Woodcock J, Givoni M, Morgan AS (2013). Health Impact Modelling of Active Travel Visions for England and Wales Using an Integrated Transport and Health Impact Modelling Tool (ITHIM). PLoS ONE.

[70] Zapata-Diomedi B, Herrera AMM, Veerman JL (2016). The effects of built environment attributes on physical activity-related health and health care costs outcomes in Australia. Health Place.

[71] Zapata-Diomedi B, Boulangé C, Giles-Corti B, Phelan K, Washington S, Veerman JL (2019). Physical activity-related health and economic benefits of building walkable neighbourhoods: a modelled comparison between brownfield and greenfield developments. Int J Behav Nutr Phys Act.

[72] Zapata-Diomedi B, Gunn L, Giles-Corti B, Shiell A, Lennert Veerman J (2018). A method for the inclusion of physical activity-related health benefits in cost-benefit analysis of built environment initiatives. Prev Med.

