# Supplementary material for: Walkability and urban built environments—a systematic review of health impact assessments (HIA)
Source: BMC Public Health. 2023 Mar 17;23:518. doi: 10.1186/s12889-023-15394-4 (PMC10024446; doi:10.1186/s12889-023-15394-4)
Supplement: Supplementary file 1 — Additional file 1: Table S1. Geographic distribution of HIAs. Table S2. Types of projects, programs or policies that is evaluated using HIA. Table S3. Type of HIA. Table S4. Type of health endpoints. Table S5. Type of data source for the HIA. Table S6. Type of results. [file 12889_2023_15394_MOESM1_ESM.docx]

# Additional File 1:

# Supplementary Result Tables S1 – S6

This file contains supplementary result tables. Please note, that for technical reasons reference numbers in this document are not identical with the reference numbers in the main article.

Table S1: Geographic distribution of HIAs

| Region/Country | Count | References |
| --- | --- | --- |
| USA | 18 | (1–18) |
| Canada | 3 | (19–21) |
| Australia | 6 | (22–27) |
| New Zealand | 1 | (28) |
| UK | 5 | (29–33) |
| Several European (incl. Germany) | 1 | (34) |
| Other European | 3 | (35–37) |
| India | 2 | (38,39) |
| Several European and Non-European countries | 1 | (40) |

Table S2: Types of projects, programs or policies that is evaluated using HIA

| Type | Count | References |
| --- | --- | --- |
| Rezoning | 1 | (1) |
| City redevelopment and new suburbs, revitalisation/regeneration of city, abandoned areas | 6 | (3,5,10,19,23,29) |
| Neighbourhood redesign | 5 | (8,20,30,36,37) |
| Scenarios/policies to support active transport * | 11 | (2,6,7,11,12,16–18,24–26,33,40) |
| Improving/extending infrastructure to facilitate active transport or public transport ** | 13 | See following lines |
| - Non-motorized transport and public transport infrastructure | 1 | (39) |
| - Bicycle and pedestrian infrastructure | 5 | (4,21,28,31,32) |
| - cycling infrastructure | 5 | (13,15,34,35,38) |
| - Public transport infrastructure | 1 | (14) |
| - Extending sidewalks | 1 | (22) |
| Other | 3 | (9,27,40) |

* Scenarios/policies refers to more general scenarios or policies to improve active transport

** infrastructure refers to more concrete projects like extending cycling networks, better sidewalks, etc,

Table S3: Type of HIA

|  | Count | References |
| --- | --- | --- |
| Quantitative | 33 | (2,3,6–16,18,19,21–28,31–40) |
| Qualitative | 4 | (4,17,20,30) |
| Quantitative and qualitative | 3 | (1,29,5) |

Table S4: Type of health endpoints

|  | Count | References |
| --- | --- | --- |
| Cardiovascular | 16 | (1,5–7,10,16,18,19,22–24,26,28,33,36,40) |
| Diabetes | 12 | (6,7,16,18,19,22–24,26,28,33,40) |
| Cancer | 8 | (5,16,22–24,26,28,33) |
| Mental illness | 6 | (5,10,16,19,28,33) |
| Premature deaths | 5 | (2,5,16,18,36) |
| All-cause mortality | 5 | (12,13,33,34,38) |
| Respiratory | 5 | (7,28,33,34,40) |
| Obesity | 4 | (1,5,7,19) |
| traffic accidents | 4 | (5,28,34,40) |

Table S5: Type of data source for the HIA

|  |  | Count | References |
| --- | --- | --- | --- |
| Primary data collection | Survey or questionnaire | 8 | (5,14,21,27,29,31,32,37) |
|  | Interview and the type (focus group/ narrative qualitative) | 7 | (29) (Semi-structured before- and-after interviews),  (19)(semi-structured),  (4)(open end),  (5)(key information interview, and focus group),  (30)(focus group),  (8)(semi-structured focus group and semi-structured in-depth interview),  38 (20)(focus group) |
|  | Observation, audit | 3 | (5,19,29) |
|  | Accelerometer | 1 | (29) |
| Secondary data use | Survey | 14 | (2,4,7–10,14,18,19,22,23,25,39,40) |
|  | group discussion | 1 | (4) |
|  | Interview | 1 | (2) |
|  | Travel diary | 1 | (2) |
| Literature review | | 5 | (3,17,24,39,40) |
| Measurement of built environment variables (GIS, ArcGIS…) | | 7 | (5,18,21,23,27,29,34) |
| Analysis of reports, inventories and other types of data | | 9 | (5,10,19,21,22,27,29,31,37) |

Table S6: Type of results

|  | Count | References |
| --- | --- | --- |
| Improvement in Health, health behaviour | 31 | (1,2,6–10,14–18,20–28,31–40) |
| Lack of changes or decrease in the health status | 3 | (7,12,30) |
| Gain in Economic value | 13 | (6,9,13,15,16,22,23,26,28,34–36,38) |
| Lack or loss of economic effect | 1 | (24) |
| Social effect | 3 | (1,10,20) |
| Policy and implementation recommendation | 6 | (3–5,8,19,39) |

References:

1. Thornton RLJ, Greiner A, Fichtenberg CM, Feingold BJ, Ellen JM, Jacky M. Jennings. Achieving a Healthy Zoning Policy in Baltimore: Results of a Health Impact Assessment of the TransForm Baltimore Zoning Code Rewrite. Public Health Rep. 2013 Nov;128(6_suppl3):87–103. DOI: 10.1177/00333549131286S313

2. Mansfield TJ, Gibson JM. Estimating Active Transportation Behaviors to Support Health Impact Assessment in the United States. Front Public Health. 2016;4:63. DOI: 10.3389/fpubh.2016.00063

3. Ross CL, Leone de Nie K, Dannenberg AL, Beck LF, Marcus MJ, Barringer J. Health Impact Assessment of the Atlanta BeltLine. American Journal of Preventive Medicine. 2012 Mar 1;42(3):203–13. DOI: 10.1016/j.amepre.2011.10.019

4. Bias TK, Abildso CG. Measuring policy and related effects of a health impact assessment related to connectivity. Preventive Medicine. 2017 Feb 1;95:S92–4. DOI: 10.1016/j.ypmed.2016.08.007

5. Hoehner CM, Rios J, Garmendia C, Baldwin S, Kelly CM, Knights DM, et al. Page Avenue health impact assessment: Building on diverse partnerships and evidence to promote a healthy community. Health & Place. 2012 Jan 1;18(1):85–95. DOI: 10.1016/j.healthplace.2011.07.005

6. MacDonald Gibson J, Rodriguez D, Dennerlein T, Mead J, Hasch T, Meacci G, et al. Predicting urban design effects on physical activity and public health: A case study. Health & Place. 2015 Sep 1;35:79–84. DOI: 10.1016/j.healthplace.2015.07.005

7. Frank LD, Fox EH, Ulmer JM, Chapman JE, Braun LM. Quantifying the health benefits of transit-oriented development: Creation and application of the San Diego Public Health Assessment Model (SD-PHAM). Transport Policy. 2022 Jan 1;115:14–26. DOI: 10.1016/j.tranpol.2021.10.005

8. Payton Foh E, Brown RR, Denzongpa K, Echeverria S. Legacies of Environmental Injustice on Neighborhood Violence, Poverty and Active Living in an African American Community. Ethn Dis. 2021;31(3):425–32. DOI: 10.18865/ed.31.3.425

9. Guo JY, Gandavarapu S. An economic evaluation of health-promotive built environment changes. Preventive Medicine. 2010 Jan 1;50:S44–9. DOI: 10.1016/j.ypmed.2009.08.019

10. Branas CC, Cheney RA, MacDonald JM, Tam VW, Jackson TD, Ten Have TR. A Difference-in-Differences Analysis of Health, Safety, and Greening Vacant Urban Space. American Journal of Epidemiology. 2011 Dec 1;174(11):1296–306. DOI: 10.1093/aje/kwr273

11. King DK, Glasgow RE, Leeman-Castillo B. Reaiming RE-AIM: Using the Model to Plan, Implement, and Evaluate the Effects of Environmental Change Approaches to Enhancing Population Health. Am J Public Health. 2010 Nov;100(11):2076–84. DOI: 10.2105/AJPH.2009.190959

12. Mansfield TJ, Rodriguez DA, Huegy J, MacDonald Gibson J. The Effects of Urban Form on Ambient Air Pollution and Public Health Risk: A Case Study in Raleigh, North Carolina. Risk Analysis. 2015;35(5):901–18. DOI: 10.1111/risa.12317

13. Gotschi T. Costs and Benefits of Bicycling Investments in Portland, Oregon. Journal of Physical Activity and Health. 2011 Jan 1;8(s1):S49–58. DOI: 10.1123/jpah.8.s1.s49

14. MacDonald JM, Stokes RJ, Cohen DA, Kofner A, Ridgeway GK. The Effect of Light Rail Transit on Body Mass Index and Physical Activity. American Journal of Preventive Medicine. 2010 Aug 1;39(2):105–12. DOI: 10.1016/j.amepre.2010.03.016

15. Gu J, Mohit B, Muennig PA. The cost-effectiveness of bike lanes in New York City. Injury Prevention. 2017 Aug 1;23(4):239–43. DOI: 10.1136/injuryprev-2016-042057

16. Nicholas W, Vidyanti I, Caesar E, Maizlish N. Routine Assessment of Health Impacts of Local Transportation Plans: A Case Study From the City of Los Angeles. Am J Public Health. 2019 Mar;109(3):490–6. DOI: 10.2105/AJPH.2018.304879

17. Perdue LA, Michael YL, Harris C, Heller J, Livingston C, Rader M, et al. Rapid health impact assessment of policies to reduce vehicle miles traveled in Oregon. Public Health. 2012 Dec 1;126(12):1063–71. DOI: 10.1016/j.puhe.2011.09.026

18. Mansfield TJ, Gibson JM. Health Impacts of Increased Physical Activity from Changes in Transportation Infrastructure: Quantitative Estimates for Three Communities. BioMed Research International. 2015;2015. DOI: 10.1155/2015/812325

19. Buregeya JM, Loignon C, Brousselle A. Contribution analysis to analyze the effects of the health impact assessment at the local level: A case of urban revitalization. Evaluation and Program Planning. 2020 Apr 1;79:101746. DOI: 10.1016/j.evalprogplan.2019.101746

20. Kaczynski AT, Sharratt MT. Deconstructing Williamsburg: Using focus groups to examine residents’ perceptions of the building of a walkable community. International Journal of Behavioral Nutrition and Physical Activity. 2010 May 27;7(1):50. DOI: 10.1186/1479-5868-7-50

21. Frank LD, Hong A, Ngo VD. Causal evaluation of urban greenway retrofit: A longitudinal study on physical activity and sedentary behavior. Preventive Medicine. 2019 Jun 1;123:109–16. DOI: 10.1016/j.ypmed.2019.01.011

22. Veerman JL, Zapata-Diomedi B, Gunn L, McCormack GR, Cobiac LJ, Mantilla Herrera AM, et al. Cost-effectiveness of investing in sidewalks as a means of increasing physical activity: a RESIDE modelling study. BMJ Open. 2016 Sep;6(9):e011617. DOI: 10.1136/bmjopen-2016-011617

23. Zapata-Diomedi B, Boulangé C, Giles-Corti B, Phelan K, Washington S, Veerman JL, et al. Physical activity-related health and economic benefits of building walkable neighbourhoods: a modelled comparison between brownfield and greenfield developments. Int J Behav Nutr Phys Act. 2019 Dec;16(1):11. DOI: 10.1186/s12966-019-0775-8

24. Zapata-Diomedi B, Herrera AMM, Veerman JL. The effects of built environment attributes on physical activity-related health and health care costs outcomes in Australia. Health & Place. 2016 Nov;42:19–29. DOI: 10.1016/j.healthplace.2016.08.010

25. Badland H, Mavoa S, Boulangé C, Eagleson S, Gunn L, Stewart J, et al. Identifying, creating, and testing urban planning measures for transport walking: Findings from the Australian national liveability study. Journal of Transport & Health. 2017 Jun 1;5:151–62. DOI: 10.1016/j.jth.2016.08.010

26. Zapata-Diomedi B, Gunn L, Giles-Corti B, Shiell A, Lennert Veerman J. A method for the inclusion of physical activity-related health benefits in cost-benefit analysis of built environment initiatives. Preventive Medicine. 2018 Jan 1;106:224–30. DOI: 10.1016/j.ypmed.2017.11.009

27. Knuiman MW, Christian HE, Divitini ML, Foster SA, Bull FC, Badland HM, et al. A Longitudinal Analysis of the Influence of the Neighborhood Built Environment on Walking for Transportation: The RESIDE Study. American Journal of Epidemiology. 2014 Sep 1;180(5):453–61. DOI: 10.1093/aje/kwu171

28. Chapman R, Keall M, Howden-Chapman P, Grams M, Witten K, Randal E, et al. A Cost Benefit Analysis of an Active Travel Intervention with Health and Carbon Emission Reduction Benefits. International Journal of Environmental Research and Public Health. 2018 May;15(5):962. DOI: 10.3390/ijerph15050962

29. Tully MA, Hunter RF, McAneney H, Cupples ME, Donnelly M, Ellis G, et al. Physical activity and the rejuvenation of Connswater (PARC study): protocol for a natural experiment investigating the impact of urban regeneration on public health. BMC Public Health. 2013 Dec;13(1):774. DOI: 10.1186/1471-2458-13-774

30. Coulson JC, Fox KR, Lawlor DA, Trayers T. Residents’ diverse perspectives of the impact of neighbourhood renewal on quality of life and physical activity engagement: Improvements but unresolved issues. Health & Place. 2011 Jan 1;17(1):300–10. DOI: 10.1016/j.healthplace.2010.11.003

31. Goodman A, Sahlqvist S, Ogilvie D. New Walking and Cycling Routes and Increased Physical Activity: One- and 2-Year Findings From the UK iConnect Study. Am J Public Health. 2014 Sep;104(9):e38–46. DOI: 10.2105/AJPH.2014.302059

32. Panter J, Heinen E, Mackett R, Ogilvie D. Impact of New Transport Infrastructure on Walking, Cycling, and Physical Activity. American Journal of Preventive Medicine. 2016 Feb 1;50(2):e45–53. DOI: 10.1016/j.amepre.2015.09.021

33. Woodcock J, Givoni M, Morgan AS. Health Impact Modelling of Active Travel Visions for England and Wales Using an Integrated Transport and Health Impact Modelling Tool (ITHIM). PLOS ONE. 2013 Jan 9;8(1):e51462. DOI: 10.1371/journal.pone.0051462

34. Mueller N, Rojas-Rueda D, Salmon M, Martinez D, Ambros A, Brand C, et al. Health impact assessment of cycling network expansions in European cities. Preventive Medicine. 2018 Apr 1;109:62–70. DOI: 10.1016/j.ypmed.2017.12.011

35. Buekers J, Dons E, Elen B, Int Panis L. Health impact model for modal shift from car use to cycling or walking in Flanders: application to two bicycle highways. Journal of Transport & Health. 2015 Dec 1;2(4):549–62. DOI: 10.1016/j.jth.2015.08.003

36. Mueller N, Rojas-Rueda D, Khreis H, Cirach M, Andrés D, Ballester J, et al. Changing the urban design of cities for health: The superblock model. Environ Int. 2020 Jan;134:105132. DOI: 10.1016/j.envint.2019.105132

37. Andersen HB, Christiansen LB, Klinker CD, Ersbøll AK, Troelsen J, Kerr J, et al. Increases in Use and Activity Due to Urban Renewal: Effect of a Natural Experiment. American Journal of Preventive Medicine. 2017 Sep 1;53(3):e81–7. DOI: 10.1016/j.amepre.2017.03.010

38. Agarwal A. Quantifying Health & Economic Benefits of Bicycle Superhighway: Evidence from Patna. Procedia Computer Science. 2021 Jan 1;184:692–7. DOI: 10.1016/j.procs.2021.03.087

39. Tiwari G, Jain D, Ramachandra Rao K. Impact of public transport and non-motorized transport infrastructure on travel mode shares, energy, emissions and safety: Case of Indian cities. Transportation Research Part D: Transport and Environment. 2016 May 1;44:277–91. DOI: 10.1016/j.trd.2015.11.004

40. Stevenson M, Thompson J, de Sá TH, Ewing R, Mohan D, McClure R, et al. Land use, transport, and population health: estimating the health benefits of compact cities. The Lancet. 2016 Dec 10;388(10062):2925–35. DOI: 10.1016/S0140-6736(16)30067-8
